# PRAME is not a frequently expressed antigen in renal cell carcinoma

**DOI:** 10.1002/bco2.70037

**Published:** 2025-05-29

**Authors:** Irvin Yi, Yael Derdikman Ofir, Jacqueline Mann, David Su, Tara Kim, Oscar Perales, Lin Zhang, Adebowale Adeniran, Harriet M. Kluger, David A. Schoenfeld

**Affiliations:** ^1^ Yale School of Medicine New Haven Connecticut USA; ^2^ Section of Medical Oncology Yale School of Medicine New Haven Connecticut USA; ^3^ Yale University New Haven Connecticut USA; ^4^ Department of Pathology Yale School of Medicine New Haven Connecticut USA

In patients with advanced renal cell carcinoma (RCC), immune checkpoint inhibitors (ICIs) have led to significant improvements in overall survival. However, not all patients respond to ICI‐based regimens, and most patients that do will eventually develop resistance.^1^ This highlights the need for complementary and alternative immunotherapeutic strategies in RCC. Many newer strategies are tumour antigen targeted, such as antibody‐drug conjugates, CAR T‐cell therapy, T‐cell receptor therapy and bispecific T‐cell engagers.^1^ Such therapies have demonstrated promising anti‐tumour activity in early‐phase trials.^1^ The identification of targetable tumour‐specific antigens is thus an important step for ICI‐resistant RCC.

Preferentially expressed antigen in melanoma (PRAME) is a protein classified under the cancer/testis antigen family. PRAME functions primarily as a repressor of retinoic acid signalling, preventing retinoic acid induced cell differentiation and proliferation arrest.^2^ It also contains multiple HLA‐specific epitopes that can be presented by MHC class I molecules, activating CD8^+^ T cells.^2^ The normal expression of PRAME is limited to testes and ovarian cells, but it is pathologically expressed in numerous solid and haematological malignancies. For example, PRAME expression is observed in 88% of primary melanomas and 95% of metastatic melanomas, 80% of non‐small cell lung cancers (NSCLCs) and 53% of breast cancers, among others.^2^ PRAME expression has been shown to be prognostic and is associated with advanced tumour stage and poor overall survival.

The combination of PRAME's restricted cancer overexpression and immunomodulatory potential have made it a promising immunotherapeutic target. PRAME‐targeting therapies have been in development for multiple malignancies using a variety of approaches, including bispecific T cell engagers, T‐cell receptor adoptive cell therapies and antibody‐drug conjugates. Ongoing trials include a Phase III trial testing a T cell receptor bispecific protein targeting PRAME and CD3 in melanoma (PRISM‐MEL‐301, NCT06112314) and Phase I/II trials enrolling PRAME‐positive patients in multiple solid tumours.^3^, ^4^ Early results from these trials have demonstrated safety and anti‐tumour activity in heavily pretreated patients across tumour types, including melanoma, ovarian cancer, head and neck cancer and synovial sarcoma.^3^, ^4^

PRAME expression patterns have not been thoroughly evaluated in RCC. A limited study found *PRAME* mRNA positivity in 15 of 39 (38%) RCC samples.^5^ A more systematic analysis of PRAME expression in epithelial tumours observed PRAME positivity by immunohistochemistry (IHC) in 20 of 175 clear cell RCC samples, with a 22% positivity rate in grade 3/4 samples versus 7% in grade 1/2 tumours.^6^

The purpose of this study was to comprehensively evaluate expression patterns of PRAME in RCC utilizing clinically annotated tissue microarrays (TMAs) of primary RCC tumours. We used an anti‐PRAME antibody (clone EPR20330, Biocare) that is an FDA‐approved in vitro diagnostic, typically used in CLIA‐certified labs to aid in immunohistochemical melanoma diagnosis. We validated the specificity of this antibody in RCC via Western blotting using a panel of RCC cell lines and known positive and negative controls. From prior studies, the melanoma cell lines MP41 and YUKRIN served as positive controls, and human granulocyte cell lysate, HUVEC (human umbilical vein endothelial cells) and BxPC3 (pancreatic cancer) cells served as negative controls.^7^ Data from the Human Protein Atlas (HPA) was also used to identify five human RCC cell lines with differential *PRAME* mRNA expression (Figure 1A).^8^ We confirmed PRAME protein expression in these cell lines to be consistent with mRNA expression patterns from the HPA, with the highest levels of expression in Caki2 and A498 cells (Figure 1B).

**FIGURE 1:**
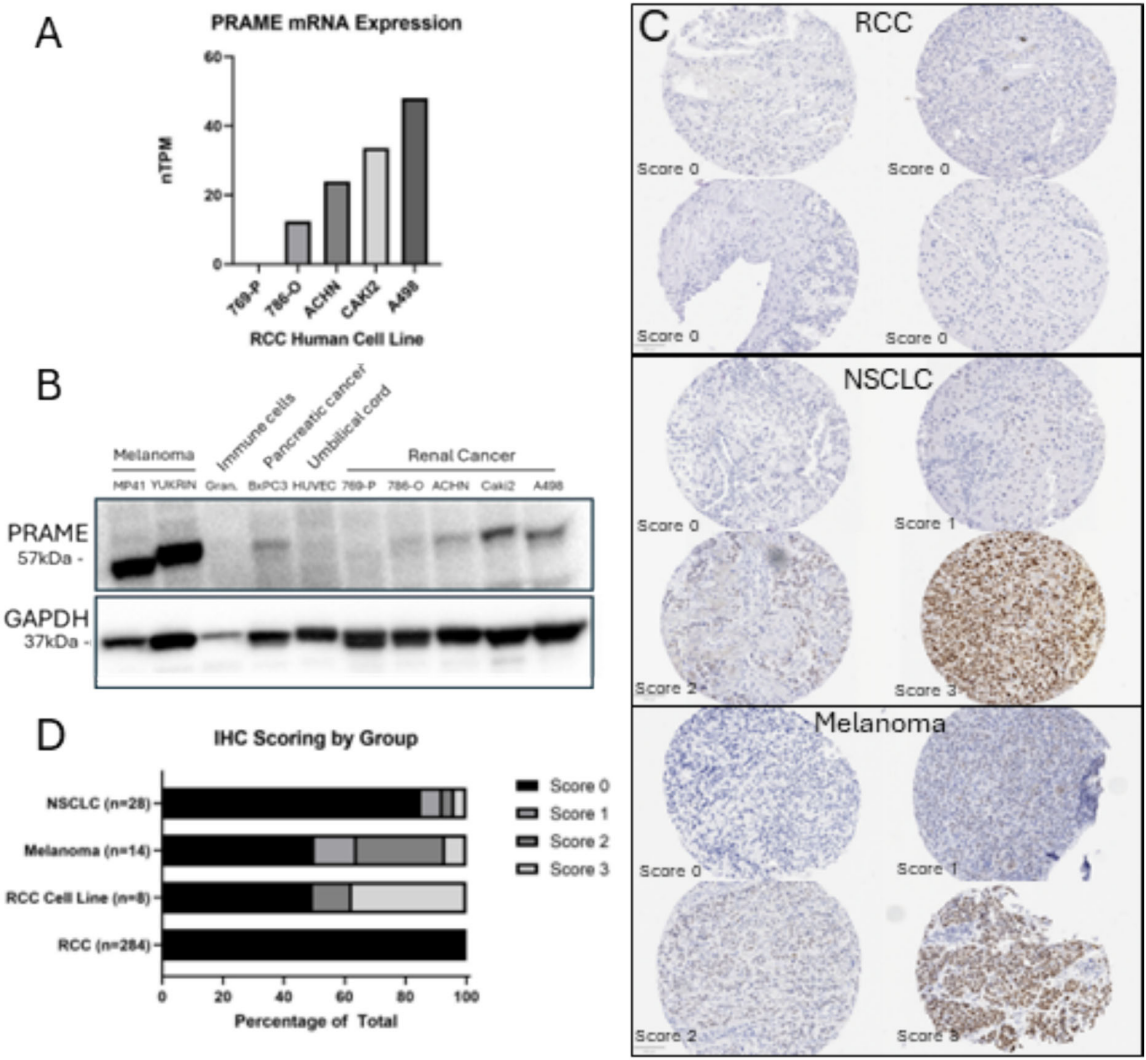
(A) RCC cell line PRAME mRNA expression (from the Human Protein Atlas). Cell lines with variable expression selected for Western blot validation. (B) Western blot of PRAME protein levels and GAPDH loading control of previously selected RCC cell lines, positive control melanoma cells, and negative control granulocytes and umbilical vein cells (HUVECs). (C) Exemplary IHC stains and scoring levels from RCC, melanoma and NSCLC cores. IHC was performed as previously described using the Leica Bond III with anti‐PRAME clone EPR20330 (Biocare) applied at 1:100. Expression was visualized by the Leica Bond polymer refine detection kit (DS9800). (D) Stacked bar graphs visualizing IHC scoring by core type.

IHC for PRAME (1:100) was performed on three TMAs: one included 285 primary RCC tumours (169 clear cell, 34 papillary, 19 oncocytoma, 7 sarcomatoid, 6 chromophobe and 10 mixed), 10 adjacent normal kidney samples and 8 RCC human cell lines; the second included 14 melanoma samples; and the third included 28 NSCLC samples. Slides were evaluated by a board‐certified pathologist (AA) and scored with a semi‐quantitative scale (0–3).

All 285 RCC primary tumour cores had a score of 0 with no demonstrated PRAME positivity (Figure 1C,D). In comparison, four of eight RCC human cell lines were PRAME positive, with one cell line having a score of 2 and three lines with a score of 3. Notably, the three RCC cell lines (A498, Caki2 and 786‐O) with PRAME levels assessed by transcriptional profiling (HPA), Western blot and IHC had consistent positive levels of PRAME expression. Seven of 14 (50%) of the melanoma tumour cores demonstrated positive staining. Of the positive cores, two of seven were scored as 1, four of seven as 2 and one of seven as 3 (Figure 1D). Four of 28 (14%) of the NSCLC tumour cores also demonstrated positive staining, with two at a score of 1, and one each at a score of 2 and 3 (Figure 1D).

This study demonstrated the absence of PRAME expression across a large cohort of 285 primary RCC tumour samples. Our findings extend prior observations in non‐clear cell RCC, which found only 1 papillary RCC specimen to be PRAME positive across 60 papillary RCC specimens, 44 oncocytomas and 20 samples of other non‐clear cell RCC subtypes.^6^ Our cohort of 34 papillary, 19 oncocytomas and 23 samples of other non‐clear cell RCC subtypes were all negative. Moreover, in our 169 clear cell RCC primary samples, we found no PRAME expression, in contrast to the study by Kaczorowski et al., in which 20 of 175 samples (11.4%) were PRAME positive.^6^ This could be due to differences in antibody choice, concentration and staining conditions, although of note both studies used FDA‐approved in vitro diagnostic antibodies. Further studies are needed to evaluate this discrepancy.

Our study had some limitations. We used a semi‐quantitative scoring system for IHC staining; quantitative methods may better elucidate lower expression levels. We also limited our investigation to primary RCC tumour specimens—it is unclear if the lack of PRAME expression in RCC extends to metastatic specimens. We also found generally lower expression of PRAME in our melanoma and NSCLC cohorts than reported in the literature, although this could be explained by our limited cohort sizes in these diseases.

In conclusion, this study is a timely inquiry into PRAME antigen expression patterns in RCC in the context of rapidly expanding PRAME‐targeted therapy development. Our findings suggest that targeting PRAME may be of limited utility in RCC. However, it is still unclear what PRAME expression threshold is sufficient for anti‐tumour activity and further characterization could yet open the door to PRAME‐directed therapies for RCC patients if low expression levels are detected.

## CONFLICT OF INTEREST STATEMENT

Dr. Harriet Kluger has received consulting fees from Iovance, Merck, Bristol‐Myers Squibb, Chemocentryx, Signatero, Gigagen, GI Reviewers, Pliant Therapeutics, Esai, Invox and Wherewolf, Teva, Replimmune, Genmab, all outside of the submitted work. HK has also received research grant funding (to Yale University) from Merck, Bristol‐Myers Squibb, Apexigen and Pfizer. The remaining authors declare that the research was conducted in the absence of any commercial or financial relationships that could be construed as a potential conflict of interest.

## ETHICS APPROVAL

Specimens and clinical information were collected with the approval of a Yale University Institutional Review Board. All authors consent to the publication of this manuscript.

## Data Availability

The dataset upon which this study is based is available upon reasonable request.
